# Admission hyperglycemia and adverse outcomes in diabetic and non-diabetic patients with non-ST-elevation myocardial infarction undergoing percutaneous coronary intervention

**DOI:** 10.1186/s12872-016-0441-x

**Published:** 2017-01-05

**Authors:** Yuanyuan Hao, Qun Lu, Tao Li, Guodong Yang, Peijing Hu, Aiqun Ma

**Affiliations:** 1 Department of Cardiovascular Medicine, First Affiliated Hospital of Xi’an Jiaotong University, Xi’an, China; 2 Shaanxi Key Laboratory of Molecular Cardiology (Xi’an Jiaotong University), Xi’an, China; 3 Key Laboratory of Environment and Genes Related to Diseases (Xi’an Jiaotong University), Ministry of Education, Xi’an, China

**Keywords:** Non-ST-elevation myocardial infarction, Diabetes mellitus, Hyperglycemia, Major adverse cardiac events

## Abstract

**Background:**

The association between admission hyperglycemia and adverse outcomes in patients with non-ST-segment elevation myocardial infarction (NSTEMI) undergoing percutaneous coronary intervention (PCI) has not been well studied, and the optimal plasma glucose cut-off values for prognosis for NSTEMI patients with and without diabetes have not been determined.

**Methods:**

According to glucose level and diabetes status, consecutive NSTEMI patients undergoing PCI (*n* = 890) were divided into four groups: without diabetes mellitus (DM) and admission plasma glucose (APG) <144 or ≥144 mg/dL; or with DM and APG <180 or ≥180 mg/dL. All patients were followed up at 30 days and 3 years after discharge, and the outcomes were assessed.

**Results:**

Admission hyperglycemia was found in 44 and 28% of the DM and non-DM patients, respectively. Multivariable analyses showed that the APG level was an independent predictor of 30-day and 3-year MACEs. Receiver operating characteristic curve analysis revealed that the appropriate cut-off values were 178 and 145 mg/dL for patients with and without DM, respectively, or 157 mg/dL for all patients.

**Conclusions:**

Admission hyperglycemia may be used to predict 30-day and 3-year MACEs in patients with NSTEMI undergoing PCI, irrespective of diabetes status. However, the optimal admission glucose cut-off values for predicting prognosis differ for patients with or without DM.

## Background

Stress hyperglycemia in patients admitted with acute myocardial infarction (AMI), with or without diabetes mellitus (DM), has been associated with major adverse cardiovascular events (MACE) and increased mortality [1–3]. However, most studies have included patients with only ST-elevation myocardial infarction (STEMI) [2, 4, 5] or included both STEMI and non-ST-elevation myocardial infarction (NSTEMI) patients [1, 3, 6–8]. STEMI and NSTEMI are the major types of AMI, and each is associated with different pathophysiological changes, complications, and prognoses [8]. Patients are usually treated with medications [1, 5–7, 9]. Therefore, it is also likely that the cutoff values for blood glucose levels at admission will differ as predictors of prognoses in these two groups.

Currently, the mainstay treatment for AMI is percutaneous coronary intervention (PCI). A number of studies have sought to establish the link between blood glucose levels at admission and adverse clinical outcomes and mortality for STEMI patients undergoing PCI. For example, Pres et al. [10] showed that elevated random glucose levels at admission were associated with higher in-hospital and long-term mortality in STEMI patients undergoing primary PCI.

Similar studies performed on NSTEMI patients undergoing primary PCI are limited (although Stefano et al. [11] showed elderly adults with DM and random admission hyperglycemia experienced higher mortality, mostly due to pre-existing cardiovascular and renal damage).

The optimal plasma glucose cut-off value for the prediction of poor outcomes in NSTEMI patients has never been determined. These values probably also differ between AMI patients with or without DM and should be calculated separately. It may be that such a cut-off value is higher in patients with DM, because they have higher average glycemia. Planer et al. [12] reported that the best predictive admission serum glucose cut-off values for 30-day mortality in STEMI patients undergoing PCI with and without DM were 231 and 149, respectively. Timmer et al. [13] determined a cut-off value of 140 mg/dL for non-DM patients with myocardial infarction.

The present study assessed the utility of admission plasma glucose (APG) for predicting MACEs both in the short term (30 days) and long term (3 years) in NSTEMI patients, with and without DM, undergoing PCI. Furthermore, the optimal cut-off APGs for predicting poor outcomes in these patients were determined.

## Methods

### Study population

The Ethics Committee of the First Affiliated Hospital of Xi’an Jiaotong University approved this retrospective study, and all participants signed informed consent forms. From January 2009 to October 2012, 890 consecutive NSTEMI patients undergoing PCI in two hospitals in China (First Affiliated Hospital of Xi’an Jiaotong University and Xi’an Central Hospital) were enrolled in this study. Patients were eligible if they had all three of the following: 1) symptoms of ischemia increasing or occurring at rest, 2) an elevated cardiac troponin I level (≥2.0 ng/mL) or troponin T level (≥0.1 ng/mL) or CK-MB (19 U/L, exceeding twice the upper limit of normal), and 3) ischemic changes assessed by electrocardiography, defined as ST-segment depression or T-wave inversion of ≥0.2 mV in two contiguous leads. Patients were excluded for having any of the following: STEMI; receiving fibrinolysis therapy; no data on glucose levels as soon as possible after admission and before the administration of any medication; bleeding history and thrombocytopenia; coronary artery bypass grafting (CABG) within 1 month; drug allergy; anticoagulation contradiction; serum creatinine >2.5 mg/dL; malignancy; or neurological deficit that limited follow-up. All patients were treated according to standard guidelines with aspirin (300 mg of chewable preparation as a loading dose, followed by 100 mg/day), clopidogrel (300 mg as a loading dose and then 75 mg/day), and unfractionated heparin (5000 U as a loading dose and then 1000 U/kg/h with a partial thromboplastin time in the range of 50–70 s). CK-MB and troponin T or I were detected in all patients.

### Date collection and variables

The following patient data were collected and analyzed: basic demographic and clinical characteristics (age, gender, hypertension, diabetes, smoker, hyperlipidemia, and pathological and therapeutic antecedents), clinical findings on admission, laboratory tests, echocardiographic changes, treatment during hospitalization, and treatment after discharge. The plasma glucose values and HbA1c levels of these patients were measured upon admission in the local laboratory at each participating center.

DM was defined as having a previous history of DM based on the medical institution standard diagnostic criteria, use of diet, oral glucose-lowering medication and/or insulin or a HbA1c ≥6.5%. This HbA1c value was recognized by the American Diabetes Association (ADA) as sufficient for the diagnosis of DM [14]. Hyperglycemia was defined as any in-hospital blood glucose measurement >140 mg/dL in accordance with the ADA consensus [15].

Admission hyperglycemia was defined as an APG concentration above the cut-off value, which discriminated between good and poor prognosis and varied over a wide range (between 6 mmol/L and 11 mmol/L) [16]. Previous studies found that the association between high APG levels and outcomes differed in individuals with and without DM [1, 3, 17, 18], and the prognostic cut-off is 144 mg/dL (8 mmol/L) for patients without DM and 180 mg/dL (10 mmol/L) for those with DM, as shown in the meta-analysis by Capes et al. [19]. Patients were divided into the following four groups: Groups 1 and 2, without DM and APG < or ≥144 mg/dL, respectively. Groups 3 and 4: with DM and APG < or ≥ 180 mg/dL.

### Outcomes measured

The primary objective of the present study of NSTEMI patients undergoing PCI was to investigate the effect of APG levels on subsequent MACEs in these patients. A MACE was defined as any of the following: re-infarction; heart failure requiring admission; target vessel revascularization (TVR) for recurrent ischemia or acute occlusion of a stent; repeated PCI or coronary artery bypass grafting (CABG); and stroke and mortality at the 30-day and 3-year follow-ups. The secondary purpose was to determine the optimal glucose cut-off values for predicting 30-day mortality in diabetic and non-diabetic NSTEMI patients undergoing PCI. Clinical follow-up variables, including re-infarction, heart failure requiring admission, and stroke and death data, were obtained from clinic visits and telephone interviews. Deaths were defined as vascular if the death certificate stated that the underlying cause of death was ischemic heart disease (I20-I25), stroke (I63, I64, and I67), or other vascular diseases (I70–I79), in accordance with the Tenth Revision of the International Classification of Diseases (ICD-10). Otherwise, deaths were defined as non-vascular.

All the patients were followed up at 30 days and 3 years after discharge. Clinical follow-up information was obtained by reviewing hospital records and conducting face-to-face interviews.

### Statistical analyses

Continuous variables were described as mean ± standard deviation or median (interquartile range), and differences among the groups were determined using Student’s *t*-test. Frequencies and proportions were reported for categorical variables and were compared using the chi-squared test. Multivariable Cox proportional hazards regression analysis was performed to identify the independent association between admission glycemia and MACEs at the 30-day or 3-year follow-ups. For all of the odds ratios (ORs), we calculated 95% confidence intervals (CIs). Kaplan–Meier methods were used to estimate the rates of cumulative occurrence of MACEs at the follow-up time points and to plot the time-to-cumulative occurrence of MACE curves. The significance of differences among the groups was determined using the log-rank test. Receiver operating characteristic (ROC) curve analyses were performed to establish the optimal cut-off values of APG for predicting 30-day mortality in patients with and without DM. All analyses were performed for the entire cohort, and the significance was established at the 0.05 level.

## Results

### Baseline characteristics

This study enrolled 890 patients, and 236 (26.5%) satisfied the criteria for DM. The median (interquartile range) APG levels were 133 (112–159) mg/dL for the entire cohort, 166 (138–202) mg/dL for patients with DM, and 124 (106–146) mg/dL for patients without diabetes.

There were no significant differences in mean age, prior myocardial infarction, prior CABG, total cholesterol, high-density lipoprotein, low-density lipoprotein, triglyceride, creatinine, number of vessels treated, and number of stents implanted in participants among the four groups (Table 1).

**Table 1:** Baseline characteristics of NSTEMI patients stratified according to diabetic status and APG

Characteristic	Participants without DM		Participants with DM	
	Group 1	Group 2	Group 3	Group 4
	APG <144 mg/dL *n* = 470	APG ≥144 mg/dL *n* = 184	APG < 180 mg/dL *n* = 133	APG ≥180 mg/dL *n* = 103
Age (years)	61.0 ± 10.8	62.3 ± 11.9	62.9 ± 10.6	63.1 ± 11.6
Female (%)	87 (18.5)^§^	39 (21.2)^§^	44 (33.1)*^#^	38 (36.9)*^#^
Hypertension (%)	249 (53.0)^§^	102 (55.4)^§^	98 (73.7)*^#^	70 (68.0)*^#^
Hyperlipidemia (%)	93 (19.8)	42 (22.8)	36 (27.1)	31 (30.1)*
Smoking (%)	273 (58.1)^§^	104 (56.5)^§^	56 (42.1)*^#^	45 (43.7)*^#^
Prior MI (%)	54 (11.5)	25 (13.6)	19 (14.3)	15 (14.6)
Prior PCI (%)	40 (8.5)^§^	24 (13)^§^	30 (22.6)*^#^	10 (9.7)^§^
Prior CABG (%)	4 (0.9)	0 (0.0)	2 (1.5)	0 (0.0)
Killip class 3–4 (%)	28 (6.0)^#^	23 (12.5)*	14 (10.5)	17 (16.5)*
Creatinine (mg/dL)	0.87 ± 0.21	0.87 ± 0.16	0.91 ± 0.24*	0.93 ± 0.29*^#^
LVEF ≤ 40% (%)	26 (5.5)	18 (9.8)	7 (5.3)	12 (11.7)*
TC (mg/dL)	162.6 ± 40.4	164.1 ± 40.2	160.0 ± 41.1	160.0 ± 44.5
TG (mg/dL)	139.5 ± 61.3	140.9 ± 66.4	141.5 ± 71.4	142.1 ± 65.1
HDL (mg/dL)	40.5 ± 11.8	39.0 ± 9.5	38.5 ± 12.5	39.8 ± 11.8
LDL (mg/dL)	98.6 ± 35.1	99.7 ± 33.8	92.3 ± 37.6	92.4 ± 39.0
Triple vessel disease or LM (%)	196 (41.7)^§^	90 (48.9)^§^	82 (61.7)*^#^	66 (64.1)*^#^
Number of vessels treated	1.45 ± 0.7	1.5 ± 0.6	1.57 ± 0.7	1.54 ± 0.7
Number of stents implanted	2.17 ± 1.3	2.33 ± 1.2	2.14 ± 1.3	2.25 ± 1.0
LAD culprit lesion (%)	203 (43.2)	91 (49.5)^§^	44 (33.1)*^#^	41 (39.8)
TIMI 0/1flow pre‐PCI (%)	153 (32.6)	52 (28.3)^§^	67 (50.4)*^#^	32 (31.1)^§^
Medication during hospitalization				
Aspirin	467 (99.4)	182 (98.9)	132 (99.2)	102 (99.0)
Ticlopidine and clopidogrel	467 (99.4)	467 (99.4)	132 (99.2)	102 (99.0)
Beta-blockers	407 (86.6)	146 (79.3)	116 (87.2)	84 (82.4)
ACEI and ARB	391 (83.2)	157 (85.3)	116 (87.2)	95 (92.2)
Statins	468 (99.6)	181 (98.4)	132 (99.2)	100 (98)
CCB	45 (9.6)	22 (12.0)	13 (12.6)	13 (10.4)
Oral hypoglycemic agent	0 (0)^#^^§^	6 (3.3)*^§^	112 (84.2)*^#^	87 (84.5)*^#^
Insulin	0 (0)^#^^§^	2 (1.1)*^§^	15 (11.3)*^#^	20 (19.4)*^#^
Medication at discharge, n (%)	453	164	120	83
Aspirin	418 (92.3)	151 (92.1)	112 (93.3)	81 (97.6)
Ticlopidine and clopidogrel	56 (12.4)	18 (11.0)	13 (10.8)	11 (13.3)
Beta-blockers	254 (56.1)	108 (65.9)	76 (63.3)	50 (60.2)
ACEI and ARB	225 (49.7)	85 (51.8)	76 (63.3)	46 (55.4)
Statins	387 (85.4)	141 (86.0)	101 (84.2)	72 (86.7)
CCB	65 (14.3)	27 (16.5)	26 (21.7)	17 (20.5)

*APG* admission plasma glucose, *MI* myocardial infarction, *PCI* percutaneous coronary intervention, *CABG* coronary artery bypass graft, *LVEF* left-ventricular ejection fraction, *TC* total cholesterol, *TG* triglyceride, *HDL* High density lipoprotein, *LDL* Low density lipoprotein, *LM* left main coronary artery, *LAD* left anterior descending, *TIMI* thrombolysis in myocardial infarction, *ACEI* angiotensin converting enzyme inhibitor, *ARB* angiotensin receptor blocker, *CCB* calcium channel blocker

*P < 0.05, vs. Group 1

^#^ *P* < 0.05, vs. Group 2

^§^ *P* < 0.05, vs. Group 3

Compared with those of Group 1, Group 2 patients had a higher rate of Killip class 3–4, patients in Group 3 had significantly fewer women and smoking patients and more patients treated with oral hypoglycemic agent and insulin during hospitalization, and Group 4 patients had significantly higher prevalences of hypertension and hyperlipidemia, a higher rate of Killip class 3–4 and LVEF ≤40%, and were more likely to be treated with an oral hypoglycemic agent and insulin during hospitalization (Table 1). Compared with those of Group 2, more patients in Group 3 and Group 4 were treated with an oral hypoglycemic agent and insulin during hospitalization (Table 1). In addition, no significant differences were observed among these groups with regard to treatment with aspirin, ticlopidine and clopidogrel, beta-blockers, ACEI and ARB, statins, and CCB during hospitalization and follow-up (Table 1).

### Influence of admission hyperglycemia on short-term MACEs

At 30 days after discharge, a higher APG was associated with significantly higher all-cause and cardiac mortality and heart failure requiring hospital admission, irrespective of diabetes status, but was not correlated with the differences in the non-cardiac mortality, rates of re-infarction, and ischemic TVR (Table 2). A significant association between the APG and rates of stroke after discharge was observed in patients without DM. In addition, the lowest early all-cause and cardiac mortality rates and the incidence of HF-required hospital admission and stroke were observed in the patients of Group 1 (i.e., without DM and APG <144 mg/dL), while the highest early all-cause and cardiac mortality rates and the incidence of HF-required hospital admission were observed in the patients of Group 4 (with DM and APG ≥180 mg/dL; Table 2).

**Table 2:** Clinical outcomes at 30 days and 3 years*

Variable Description	Participants without diabetes mellitus		Participants with diabetes mellitus	
	Group1	Group 2	Group3	Group 4
	APG < 44 mg/dL *n* = 470	APG ≥144 mg/dL *n* = 184	APG < 180 mg/dL *n* = 133	APG ≥180 mg/dL *n* = 103
30-day outcomes (%)				
Death, all-cause	2 (0.4)*^§^^+^	7 (3.8)*^§^^+^	3 (2.3)^#^^§^^+^	9 (8.7)^#^^§^^+^
Cardiac	2 (0.4)*^§^^+^	6 (3.3)*^§^^+^	2 (1.5)^#^^§^^+^	8 (7.8)^#^^§^^+^
Non-cardiac	0 (0.0)	1 (0.5)	1 (0.8)	1 (1.0)
Reinfarction	0 (0.0)	1 (0.5)	1 (0.8)	1 (1.0)
HF required hospital admission	3 (0.6)*^§^^+^	5 (2.7)*^§^^+^	2 (1.5)^#^^§^^+^	6 (5.8)^#^^§^^+^
Ischemic TVR	0 (0.0)	0 (0.0)	0 (0.0)	0 (0.0)
Stroke	0 (0.0)*^§^	2 (1.1)*^§^	1 (0.8)^§^	1 (1.0)^§^
3-year outcomes (%)				
Death, all-cause	17 (3.6)*^§^^+^	20 (10.9)*^§^+	13 (9.8)^#^^§^^+^	20 (19.4)^#^^§^^+^
Cardiac	14 (3.0)*^§^^+^	17 (9.2)*^§^^+^	12 (9.0)^#^^§^^+^	19 (18.4)^#^^§^^+^
Non-cardiac	3 (0.6)	3 (1.6)	1 (0.8)	1 (1.0)
Reinfarction	5 (1.1)*^§^^+^	8 (4.3)*^§^^+^	5 (3.8)^§^^+^	9 (8.7)^§^^+^
HF required hospital admission	16 (3.4)*^§^^+^	22 (12.0)*^§^^+^	15 (11.3)^§^^+^	18 (17.5)^§^^+^
Ischemic TVR	3 (0.6)	2 (1.1)	2 (1.5)	2 (1.9)
Stroke	4 (0.9)^§^^+^	5 (2.7)^§^^+^	2 (1.5)^§^^+^	5 (4.9)^§^^+^

*APG* admission plasma glucose, *HF* heart failure, *TVR* target lesion revascularization

*P < 0.05, Group 1 vs. Group 2

^#^ *P* < 0.05, Group 3 vs. Group 4

^§^ *P* < 0.05, Group 1 vs. other three groups

^+^ *P* < 0.05, Group 4 vs. other three groups

### Influence of admission hyperglycemia on long-term MACEs

An association between APG and MACEs was observed early after the acute event and remained stable through 3 years of follow-up (Table 2). Higher APG was associated with a significantly higher all-cause and cardiac mortality in patients irrespective of diabetes status, but was not associated with non-cardiac mortality or rates of ischemic TVR or stroke. In patients without DM, a significant association between APG and rates of re-infarction or heart failure requiring hospital admission was observed. The lowest late rates of all-cause and cardiac mortality re-infarction and heart failure requiring hospital admission and stroke were observed in Group 1 (without DM and APG <144 mg/dL), while the highest were observed in Group 4 (with DM and APG ≥180 mg/dL).

### Survival analysis

**Fig. 1:**
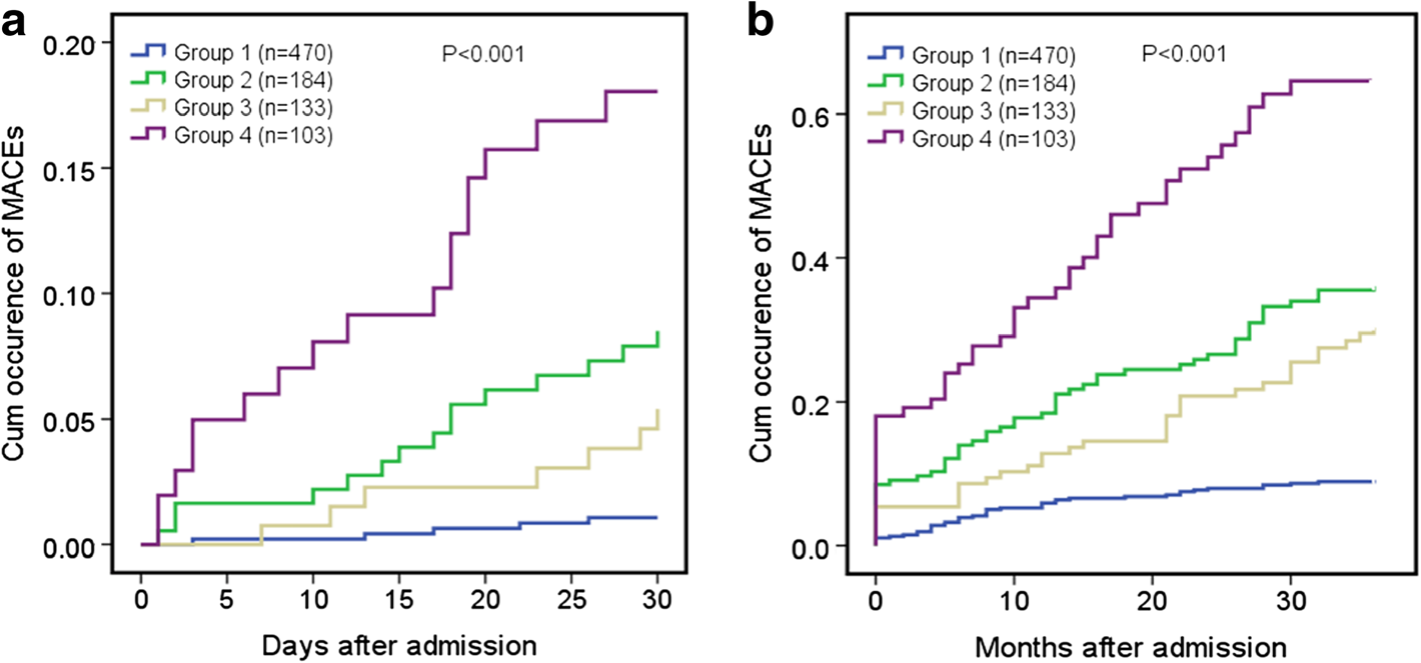
The 30-day (**a**) and 3-year (**b**) cumulative occurrence of MACEs for curves in NSTEMI patients undergoing PCI stratified according to diabetic status and admission plasma glucose (APG). Patients in Group 2 had higher cumulative occurrence rates of 30-day and 3-year MACEs. Patients in Group 4 had higher cumulative occurrence rates of 30-day and 3-year MACEs. Groups 1 and 2: without DM, APG <144 or ≥ 144 mg/dl. Groups 3 and 4: with DM, APG < or ≥ 180 mg/dl

The Kaplan–Meier survival curves showed that, compared with Group 1 (without DM, APG <144 mg/dL), Group 2 (without DM, APG ≥144 mg/dL) patients had higher cumulative rates of MACEs at 30 days and 3 years (Fig. 1). Compared with those in Group 3 (DM, APG <180 mg/dL), patients in Group 4 (DM, APG ≥180 mg/dL) had higher cumulative MACE rates at 30 days and 3 years. When stratified by diabetes status and the APG levels, the highest 30-day and 3-year cumulative MACE rate was observed in Group 4, while the lowest was that of Group 1.

### Optimal cut-off values

**Fig. 2:**
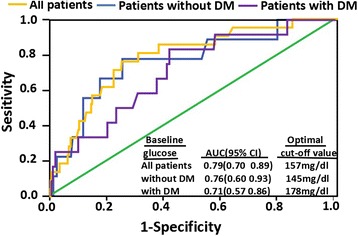
ROC curves for APG mortality in all patients, with and without diabetes

Multivariate analysis revealed that an elevated APG was an independent predictor of 30-day and 3-year MACEs, irrespective of diabetes status (Table 3). By the ROC analysis, the optimal cut-off values for predicting 30-day mortality were 157 mg/dL for the entire cohort; 145 mg/dL for patients without DM; and 178 mg/dL for patients with DM, with areas under the curve of 0.79, 0.76, and 0.71, respectively (Fig. 2).

**Table 3:** Multivariable predictors of MACEs at 30 days and 3 years stratified by diabetes status

	Variable	B	Adjusted HR [95%CI]	*P* value
30-day MACE				
All patients	APG	0.016	1.016 [1.012, 1.020]	<0.001
	Creatinine	1.243	3.466 [1.121,10.716]	0.031
	Age	0.33	1.034 [1.004,1.065]	0.028
	Prior MI	1.442	4.229 [2.314,7.731]	<0.001
Patients without DM	APG	0.017	1.018 [1.009,1.027]	<0.001
	Prior MI	1.714	5.552 [2.211, 13.940]	<0.001
	Age	0.046	1.047 [1.004,1.092]	0.032
Patients with DM	APG	0.014	1.014 [1.008,1.020]	<0.001
	Prior MI	1.338	3.813 [1.648,8.819]	0.002
	Killip class 3-4	1.186	3.273 [1.299,8.246]	0.012
	Prior PCI or CABG	1.728	5.628 [2.285,13.857]	<0.001
3-year MACEs				
All patients	APG	0.013	1.013 [1.010,1.015]	<0.001
	LVEF	−0.20	0.980 [0.967,0.994]	0.004
	Triple vessel disease or LM	0.452	1.572 [1.149,2.151]	0.005
	Beta-blockers	−0.417	0.659 [0.465,0.935]	0.019
	Prior MI	0.558	1.748 [1.212,2.520]	0.003
	Age	0.014	1.014 [1.000,1.028]	0.043
Patients without DM	APG	0.017	1.017 [1.013,1.021]	<0.001
	LVEF	−0.029	0.972 [0.954,0.990]	0.002
	Triple vessel disease or LM	0.815	2.259 [1.493,3.420]	<0.001
	Hypertension	0.480	1.617 [1.068,2.448]	0.023
Patients with DM	APG	0.009	1.009 [1.006,1.013]	<0.001
	Prior MI	1.017	2.765 [1.690,4.525]	<0.001
	Killip class 3-4	0.637	1.891 [1.090,3.282]	0.024
	Creatinine	0.961	2.614 [1.279,5.341]	0.008

*APG* admission plasma glucose, *MACE* major adverse cardiac event, *DM* diabetes mellitus. Other abbreviations as in Table 1

## Discussion

The present study investigated the APG as an indicator of the risk of short- and long-term MACEs in diabetic and non-diabetic NSTEMI patients undergoing PCI reperfusion. The analysis determined that, irrespective of diabetic status, APG could be used to predict 30-day and 3-year MACEs. Secondly, the predictive optimal admission hyperglycemia cut-off values for 30-day mortality were 157 mg/dL for all patients (area under the curve [AUC], 0.79), 178 mg/dL for those with DM (AUC, 0.71), and 145 mg/dL for patients without DM (AUC, 0.76).

Hyperglycemia on admission has been considered an acute stress response [20, 21], particularly in STEMI patients with severe left ventricular dysfunction [22]. Some studies purport that hyperglycemia on admission is causally linked to poor outcomes after AMI or is simply an epiphenomenon related to the severity of disease. Most recent studies have reported that admission hyperglycemia after AMI is causally linked to further deterioration due to myocardial damage and increased risk of death in patients, with or without diabetes. [1–9, 12]. Yet, the clinical and prognostic significance of high APG levels have differed between STEMI and NSTEMI patients [8]. Since several similar studies have been conducted on STEMI patients [2, 4, 5], our study focused on NSTEMI patients undergoing PCI, to explore the effects of APG on the rate of after hospital discharge, and its prognostic value in the absence or presence of DM.

The mechanisms by which hyperglycemia causes unfavorable effects in patients with AMI are not understood well. However, several studies have suggested that a wide variety of pathophysiologic changes linked to hyperglycemia contribute to increased mortality and morbidity rates. It is notable that acute hyperglycemia may exert significant hemodynamic effects even in normal subjects. A previous study showed that plasma glucose levels maintained at 15.0 mmol/L for 2 h in healthy subjects significantly increased the mean heart rate (+9 beats per minute), systolic and diastolic blood pressures (+20 and +14 mmHg, respectively), and plasma catecholamine levels. These hemodynamic effects were abolished by glutathione, a widely recognized free radical scavenger [23], suggesting that these changes were mediated by an oxidative-linked mechanism [24].

Stress hyperglycemia after AMI has been be associated with endothelial and microvascular dysfunction induced by oxidative stress and amplified inflammatory immune reactions. Esposito et al. [25] found that circulating inflammatory cytokine levels closely correlated with plasma glucose levels, that is, increasing as plasma glucose levels increased, and immediately returning to normal as the plasma glucose level decreased to normal. Increased serum inflammatory cytokine concentrations may intensify oxidative stress and further damage endothelial function, while reductions in circulating levels of inflammatory cytokines were accompanied by improved endothelial function [26].

In addition, some studies have suggested that the prothrombotic state generated by hyperglycemia reduces plasma fibrinolytic activity and activated tissue plasminogen [27]. Through these mechanisms, stress hyperglycemia after AMI is closely linked to impaired myocytes [27, 28], impaired left ventricular function and exacerbated cardiac damage induced by acute ischemia/reperfusion injury [29].

In conclusion, acute hyperglycemia induced by oxidative stress, inflammation, apoptosis, endothelial dysfunction, hypercoagulation, platelet aggregation, and impairment of ischemic preconditioning damages the ischemic myocardium. In the present study, an APG >140 mg/dL (7.8 mmol/L) was defined as admission hyperglycemia in accordance with the 2013 ADA Standard of Medical Care criteria 15, which were determined based on a number of large scale studies of the effect of glucose levels on mortality [30–33]. Our study confirmed that APG scores positively correlated with rates of MACEs in both diabetic and non-diabetic patients.

Our study also determined the optimal cut-off APG value for predicting short-term mortality in NSTEMI patients undergoing PCI. For assessing prognosis, stress hyperglycemia can be defined as an APG above the cut-off value. Such a cut-off value, determined via a ROC curve, should differentiate patients who achieve an uneventful recovery from those with poor outcomes during the observation period. In addition, for AMI patients with DM, the prognostic cut-off value for hyperglycemia on admission should not be the same as for non-diabetic AMI patients, because the former have a higher average blood glucose level [34]. Indeed, we found in the present study that 178 mg/dL was the optimal cut-off point for patients with DM and 145 mg/dL for the patients without DM. To the best of our knowledge, our study is the first to analyze separately the optimal cut-off APGs for NSTEMI patients with and without DM, to predict short-term mortality. Finally, our results suggest that elevated glucose levels may be an important biomarker of increased MACEs in both diabetic and non-diabetic patients.

### Study limitations

The statistical power of the present study was limited by the small number of patients in some of the groups. Also, we were not able to collect data regarding liver failure, obesity, physical activity, inflammatory markers, or socioeconomic status, and therefore, we were not able to adjust the analysis for these potential confounders. Finally, although herein we reveal that hyperglycemia on admission was associated with unfavorable short- and long-term outcomes of both diabetic and non-diabetic NSTEMI patients undergoing PCI treatment, the effects of treating hyperglycemia after admission were not assessed. Our results warrant corroboration with a study of larger scale.

## Conclusions

An elevated APG level, regardless of the diagnosis of diabetes, results in higher short-term and long-term poor outcomes in patients with NSTEMI undergoing PCI. The optimal cut-off values for blood glucose in the prognosis of patients with and without DM are different.

## Abbreviations

ACEI: Angiotensin-converting enzyme inhibitor
ADA: American Diabetes Association
AMI: Acute myocardial infarction
APG: Admission plasma glucose level
ARB: Angiotensin receptor blocker
AUC: Area under curve
CABG: Coronary artery bypass grafting
CAD: Coronary artery disease
CCB: CALCIUM channel blocker
CI: Confidence interval
CK-MB: Creatine kinase-myocardial band
cTnI: Cardiac troponin I
DM: Diabetes mellitus
ECG: Electrocardiograph
HDL: High density lipoprotein
HF: Heart failure
HR: Hazard ratio
IQR: Interquartile range
LAD: Left anterior descending
LDL: Low density lipoprotein
LM: Left main coronary artery
LVEF: Left-ventricular ejection fraction
MACEs: Major adverse cardiac events
MI: Myocardial infarction
NSTEMI: Non-ST-elevation myocardial infarction
OR: Odds ratios
PCI: Percutaneous coronary intervention
ROC: Receiver operator characteristic
RR: Relative risk
SD: Standard deviation
STEMI: ST-segment elevation myocardial infarction
TC: Serum total cholesterol
TG: Triglyceride
TIMI: Thrombolysis in myocardial infarction
TVR: Target vessel revascularization
